# Association between triglyceride-to-high-density lipoprotein cholesterol ratio and prediabetes: a cross-sectional study in Chinese non-obese people with a normal range of low-density lipoprotein cholesterol

**DOI:** 10.1186/s12967-022-03684-1

**Published:** 2022-10-22

**Authors:** Liling Wu, Xiaodan Wu, Haofei Hu, Qijun Wan

**Affiliations:** 1 Department of Nephrology, The First Affiliated Hospital of Shenzhen University, Shenzhen, 518000 Guangdong Province China; 2 Department of Nephrology, Shenzhen Second People’s Hospital, No.3002 Sungang Road, Futian District, Shenzhen, 518000 Guangdong Province China; 3 Department of Anesthesiology, Nanfang Hospital, Southern Medical University, Guangzhou, 510515 Guangdong People’s Republic of China

**Keywords:** Prediabetes, Triglyceride-to-high-density lipoprotein cholesterol ratio, Non-linear relationship

## Abstract

**Background:**

Evidence about the relationship between triglyceride-to-high-density lipoprotein cholesterol (TG/HDL-C) ratio and prediabetes (Pre-DM) in Chinese non-obese people with a normal range of low-density lipoprotein cholesterol (LDL-c) is limited. Therefore, the present study was undertaken to explore the link of the TG/HDL-C ratio on Pre-DM among non-obese Chinese population with a normal range of LDL-c.

**Methods:**

This study was a cross-sectional study that enrolled 153163 non-obese individuals with a normal range of low-density lipoprotein cholesterol in a Chinese hospital from January 2010 to December 2014. Logistic regression model, generalized additive model (GAM), smooth curve fitting and a series of sensitivity analyses was used to evaluate the association between TG/HDL-C ratio and Pre-DM.

**Result:**

The prevalence of Pre-DM was 9.77%.The median TG/HDL-C ratio was 0.671 (interquartile range, 0.468–1.010). After adjusting covariates, the results showed that TG/HDL-C ratio was positively associated with Pre-DM ((OR = 1.185, 95%CI 1.145–1.226). In addition, the TG/HDL-C ratio level has a non-linear relationship with the incidence of Pre-DM, in which the inflection point was 1.617. The effect sizes (OR) on the left and right sides of the inflection point were 1.312 (95%CI 1.242–1.386) and 0.980 (95%CI 0.898–1.070), respectively. And the sensitive analysis demonstrated the robustness of the results. Subgroup analysis showed a stronger association between TG/HDL-C ratio and Pre-DM in females and the population with 30 years  < age  < 40 years, 18.5 kg/m^2^ < body mass index  < 24 kg/m^2^, and ALT < 40U/L.

**Conclusion:**

This study demonstrates a positive and non-linear relationship between TG/HDL-C ratio and Pre-DM in Chinese non-obese people with a normal range of low-density lipoprotein cholesterol. TG/HDL-C ratio is strongly related to Pre-DM when TG/HDL-C ratio is less than 1.617. It makes sense to reduce the TG/HDL-C ratio level below the inflection point from a treatment perspective.

**Supplementary Information:**

The online version contains supplementary material available at 10.1186/s12967-022-03684-1.

## Background

Prediabetes (Pre-DM) means the blood sugar level is higher than normal, but not yet in the diabetic range [1]. Pre-DM affects about 35.7% of adults in China [2], with an annual conversion rate of Pre-DM to diabetes (DM) of 5–10% [3]. The treatment of DM can prevent some devastating complications, but it does not restore normoglycemiam (NG) or eliminate all adverse consequences. Furthermore, Pre-DM increases the risk of developing DM, but it does not always progress to DM [4]. The Diabetes Prevention Program Research Group pointed out that progression of Pre-DM to DM can be prevented by lifestyle changes as well as medications [5]. Therefore, screening for the risk factors of developing Pre-DM and treat these conditions earlier rather than later to prevent progression of disease and adverse outcomes.

Dyslipidemia is characterized by abnormal traditional lipid parameters, such as elevated total cholesterol (TC), triglyceride (TG), low-density lipoprotein cholesterol (LDL-C) levels, and decreased high-density lipoprotein cholesterol (HDL-C) levels, which predict the onset of Pre-DM [6]. In National Health and Nutrition Examination Surveys 2011–2014, > 50% adults with Pre-DM had dyslipidemia [7]; However, global guidelines advocate aggressive lipid management in type 2 diabetes (T2DM) but most have not asserted a similar position for Pre-DM [8]. To date, previous study including 2680 participants in China suggested that triglyceride-to-high-density lipoprotein cholesterol (TG/HDL-C) ratio was positively related to the Pre-DM (OR = 3.445, 95% CI 2.417–4.921) after adjusting for confounders [9]. Unfortunately, neither study performs subgroup analyses nor explores the non-linear relationship between TG/HDL-C ratio and Pre-DM. In addition, the study is also limited by the small sample size.

Obesity is the risk factor for the development of Pre-DM. However, previous study showed that a significant proportion (13%) in the study of 254 non-obese young adults had prediabetes [10].

Since prediabetes appears to be prevalent in non-obese adults, it is particularly important to screen risks factors for this population. In addition, traditional lipid parameters including TG, HDL-C, LDL-C and TC, previous study showed the high level of LDL-C influenced the onset of Pre-DM [8]. However, participants with elevated TG levels and lower HDL-C levels also promoted the onset of prediabetes [8, 11]. A study of TG/HDL-C ratio and Pre-DM in non-obese Chinese with a normal range of low-density lipoprotein cholesterol is limited.

Therefore, we postulated that the TG/HDL-C ratio level might be associated with the incident Pre-DM in non-obese Chinese with a normal range of low-density lipoprotein cholesterol. To test this hypothesis, a retrospective cross-sectional study with 153163 individuals was performed to explore the relationship between TG/HDL-C ratio levels and Pre-DM. A logistic regression model, smooth curve fitting and a series of sensitivity analyses was used to evaluate the association between TG/HDL-C ratio and Pre-DM.

## Methods

### Study design and population

This cross-sectional study included individuals at Wenzhou Medical Center, Wenzhou People’s Hospital, China, from January 2010 to December 2014. The raw data was downloaded freely from the DATADRYAD database (www.datadryad.org) provided by Sun et al [12]. This research was conducted under the approval of the Clinical Research Ethics Committee in Wenzhou People’s Hospital [12].

**Fig.1:**
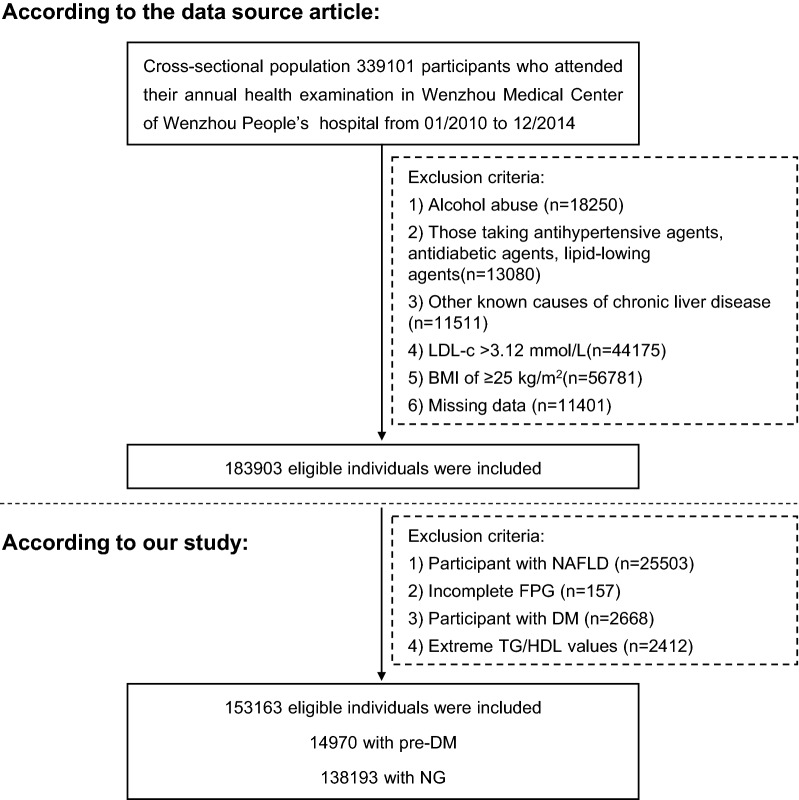
Study design and participant flow

The original research initially enrolled 339101 individuals [12]. Exclusion criteria included excessive alcohol consumption [12], chronic liver disease, body mass index (BMI)  ≥ 25 kg/m^2^ or LDL-C  > 3.12 mmol/L, patients taking antihypertensive, lipid-lowering or hypoglycemic drugs, those with missing data on HDL-C, TG, and FBG, patients with nonalcoholic fatty liver disease (NAFLD) or DM, and outliers in the TG/HDL ratio [13, 14]. 185938 participants were excluded (Fig. 1). Multiple imputations were used to handle the missing data of covariates including alanine aminotransferase (ALT), γ-glutamyl transpeptidase (GGT), aspartate aminotransferase (AST), blood urea nitrogen (BUN), albumin (ALB), globulin (GLB), direct bilirubin (DBIL) and total bilirubin (TB).

### Variables

#### TG/HDL-C ratio

The TG/HDL-C ratio was treated as a continuous variable. TG/HDL-C ratio = TG divided by HDL-C.

#### Outcomes

The interesting outcome variable was Pre-DM (dichotomous variable: 1 = Pre-DM, 0 = NG). Diabetic status includes NG (FPG < 5.6 mmol/L or HbA1c < 5.7%), Pre-DM (5.6 ≤ FPG ≤ 6.9 mmol/L or 5.7% ≤ HbA1c ≤ 6.4%), T2DM (FPG ≥ 7.0 mmol/L or HbA1c ≥ 6.5%) [7]. The World Health Organization had defined obesity in Asians as BMI ≥ 25 kg/m^2^; non-obese persons with BMI < 25 kg/m^2^ [2, 12, 15, 16].

#### Covariates

The following variables were used as covariates: (1) continuous variables: age, BMI, ALB, ALT, AST, fasting plasma glucose (FBG), BUN, creatinine (Cr), uric acid (UA), TC, TG, HDL-C, DBIL, TB, GGT, GLB and LDL-C; (2) categorical variables: sex. All the biochemical values were measured by an automated analyzer. Data were collected under standardized conditions and processed according to uniform procedures [12].

### Statistical analysis

Participants were categorized by quintile of the TG/HDL-C ratio. Continuous variables were expressed as mean ± standard deviation (SD) or median (interquartile ranges: IQR). Categorical variables were expressed as percentages. χ2 (categorical variables), One-Way ANOVA test (normal distribution), or Kruskal-Whallis H test (skewed distribution) were used to test for differences among different TG/HDL-C ratio groups. Prevalence rates were expressed in cumulative prevalence.

The association between TG/HDL-C ratio and Pre-DM was investigated using multiple logistic regression analyses, controlling for the important confounding variables, including age, sex, BMI, ALT, AST, GGT, ALB, GLB, DBIL, BUN, Scr, UA, TC, LDL-C, TB (model I and model II). Effect sizes (odds ratio: OR) with 95% confidence intervals (CI) were recorded. Besides, we also used a GAM to insert the continuity covariate into the equation (model III) as a curve to ensure the robustness of the results. Besides, when exploring the association between TG/HDL-C ratio and Pre-DM in other sensitivity analyses, we excluded participants with LDL-C  < 2.07 mmol/L or TG > 1.7 mmol/L.

Subgroup analyses were conducted to assess the consistency of the association between TG/HDL-C ratio and the incident Pre-DM in subgroups defined by age (< 30, ≥ 30 to < 40, ≥ 40 to < 50, ≥ 50 to < 60, ≥ 60 to < 70, ≥ 70 years), gender, BMI (< 18.5, ≥ 18.5 to < 24, ≥ 24 kg/m2), ALT (≤ 40, > 40U/L) and GLB (< 30, ≥ 30 g/L).

Smooth curve fitting and GAM were used to demonstrate the association of TG/HDL-C ratio with Pre-DM. The inflection point and threshold effect of TG/HDL-C ratio on event Pre-DM was calculated using a two-piece linear regression model.

Statistical analysis was conducted using the statistical packages R (http://www.R-project.org, The R Foundation) and EmpowerStats (http://www.empowerstats.com, X&Y Solutions, Inc, Boston, MA). P values < 0.05 (two-sided) were considered statistically significant.

## Results

### Characteristics of participants

153163 adults were included in the final analysis (Fig. 1), mean age was 41 years and 67302 (49.94%) participants were male. The median TG/HDL-C ratio was 0.671 (interquartile range, 0.468–1.010). The prevalence of Pre-DM was 9.77% (14970/153163). The participants were categorized by TG/HDL-C ratio quintiles (Q1: < 0.4, Q2: ≥ 0.4– < 0.6, Q3: ≥ 0.6– < 0.8, Q4: ≥ 0.8– < 1.1, Q5: ≥ 1.1). We grouped participants by TG/HDL-C ratio quintiles. Notable differences between the TG/HDL-C ratio groups were seen in the demographic and clinical characteristics. Compared with the Q1 group, participants with higher TG/HDL-C ratio had the higher value of BMI, GGT, ALB, ALT, UA, AST, TC, BUN, LDL-C and a higher proportion of men (Table 1).

**Table 1:** The baseline characteristics of participants

TG/HDL-C Quintile	Q1(< 0.4)	Q2(≥ 0.4– < 0.6)	Q3(≥ 0.6– < 0.8)	Q4(≥ 0.8– < 1.1)	Q5(≥ 1.1)	*P*-value
N	30631	30631	30626	30631	30644	
Age(years)	36.6 ± 12.0	38.0 ± 13.0	39.3 ± 13.8	40.8 ± 14.3	43.2 ± 14.3	< 0.001
Gender						< 0.001
Female	24953 (81.5%)	21099 (68.9%)	17684 (57.7%)	13362 (43.6%)	8763 (28.6%)	
Male	5678 (18.5%)	9532 (31.1%)	12942 (42.3%)	17269 (56.4%)	21881 (71.4%)	
BMI(kg/m^2^)	20.2 ± 1.9	20.6 ± 2.0	21.0 ± 2.1	21.4 ± 2.0	22.2 ± 1.8	< 0.001
GGT(U/L)	16.4 (13.0–24.0)	18.0 (14.0–25.0)	19.0 (15.0–27.0)	21.0 (16.0–30.8)	25.0 (19.0–38.0)	< 0.001
ALT(U/L)	14.0 (10.0–20.0)	14.0 (11.0–20.6)	15.0 (11.0–22.0)	16.0 (12.0–23.6)	19.0 (14.0–26.0)	< 0.001
AST(U/L)	20.0 (17.0–24.0)	20.0 (17.0–24.0)	20.0 (17.0–24.4)	21.0 (18.0–25.0)	22.0 (18.8–26.0)	< 0.001
ALB(g/L)	44.4 ± 3.0	44.4 ± 3.0	44.5 ± 3.1	44.6 ± 3.0	44.8 ± 3.0	< 0.001
GLB(g/L)	29.1 ± 3.7	29.3 ± 3.8	29.4 ± 3.8	29.4 ± 3.8	29.4 ± 3.8	0.001
TB(umol/L)	12.1 ± 5.1	12.0 ± 5.0	12.1 ± 5.0	12.1 ± 5.1	12.1 ± 5.1	< 0.001
DBIL(umol/L)	1.9 (1.4–2.6)	1.9 (1.4–2.6)	1.9 (1.3–2.6)	1.8 (1.3–2.5)	1.8 (1.2–2.5)	< 0.001
BUN(mmol/L)	4.3 ± 1.2	4.3 ± 1.2	4.3 ± 1.3	4.4 ± 1.3	4.5 ± 1.3	< 0.001
Scr(umol/L)	70.6 ± 14.7	74.1 ± 18.1	77.0 ± 22.9	80.6 ± 22.6	84.6 ± 26.3	< 0.001
UA(umol/L)	231.9 ± 68.0	249.0 ± 74.6	266.3 ± 78.4	288.7 ± 81.8	321.2 ± 85.5	< 0.001
FPG(mmol/L)	4.9 ± 0.4	5.0 ± 0.4	5.0 ± 0.4	5.1 ± 0.5	5.1 ± 0.5	< 0.001
TC(mmol/L)	4.5 ± 0.7	4.4 ± 0.7	4.4 ± 0.7	4.5 ± 0.7	4.6 ± 0.7	< 0.001
TG(mmol/L)	0.6 (0.5–0.7)	0.8 (0.7–0.9)	1.0 (0.9–1.1)	1.2 (1.1–1.4)	1.8 (1.5–2.2)	< 0.001
HDL-C(mmol/L)	1.8 ± 0.3	1.6 ± 0.3	1.5 ± 0.3	1.3 ± 0.2	1.2 ± 0.2	< 0.001
LDL-C(mmol/L)	2.0 ± 0.5	2.1 ± 0.5	2.2 ± 0.5	2.3 ± 0.5	2.4 ± 0.4	< 0.001
TG/HDL-C	0.4 (0.3–0.4)	0.5 (0.5–0.5)	0.7 (0.6–0.7)	0.9 (0.8–1.0)	1.5 (1.3–1.9)	< 0.001

Values are n (%) or mean ± SD or median (quartile)

*ALB* albumin, *ALT* alanine aminotransferase, *AST* aspartate aminotransferase, *BMI* body mass index, *BUN* blood urea nitrogen, *Scr* serum creatinine, *DBIL* direct bilirubin, *TB* total bilirubin, *FPG* fasting plasma glucose, *GGT* γ-glutamyl transpeptidase, *GLB* globulin, *HDL-C* high-density lipoprotein cholesterol, *LDL-C* low-density lipoprotein cholesterol, *TC* total cholesterol, *TG* triglyceride, *UA* uric acid, *TG/HDL-C* triglyceride-to-high-density lipoprotein cholesterol ratio

**Fig.2:**
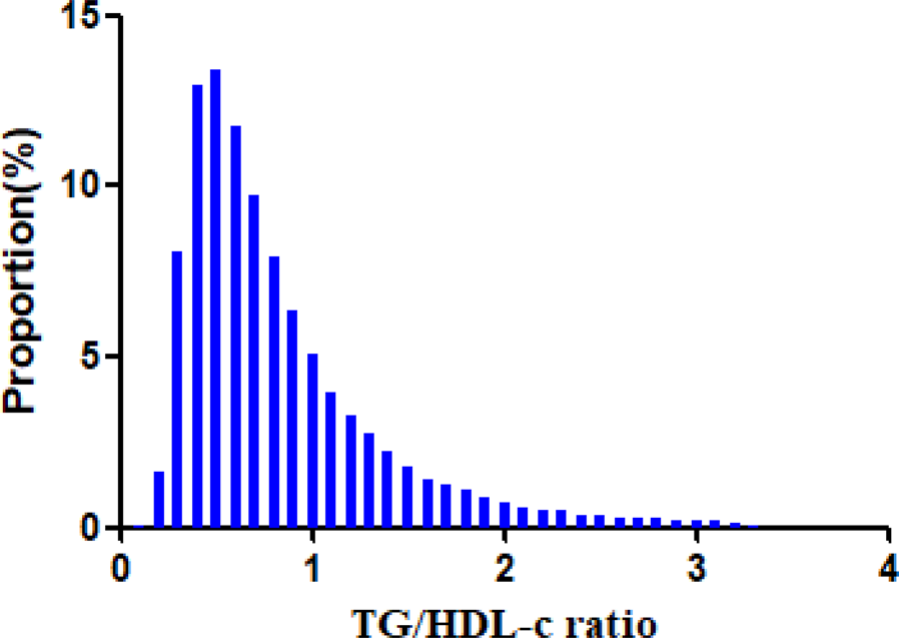
Distribution of TG/HDL-C ratio. It presented a skewed distribution while being in the range from 0.108 to 3.290

**Fig.3:**
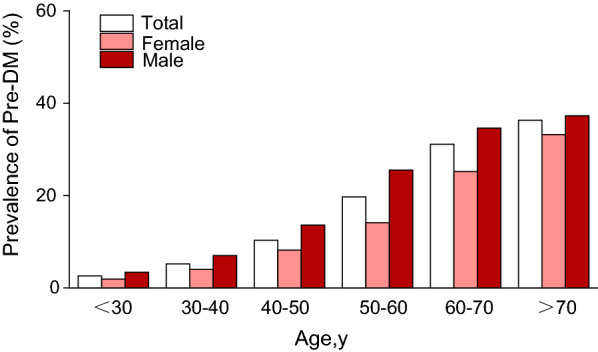
Pre-DM prevalence of age stratification by 10 intervals. In age stratification by 10 intervals, the prevalence of Pre-DM increases with age and is more common in men than women

Figure 2 showed that the distribution of TG/HDL-C ratio levels was normally distributed, ranging from 0.108 to 3.290, with a mean of 0.824. In age stratification by 10 intervals, the prevalence of Pre-DM increased with age and was more common in men than women (Fig. 3).

### Marked elevation in the TG/HDL-C ratio level in participants with Pre-DM

**Fig.4:**
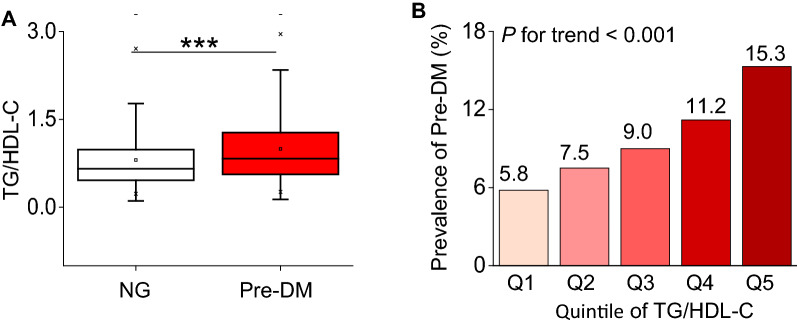
Elevation of TG/HDL-C and its relationship with incident Pre-DM. **A** Levels of TG/HDL-C in baseline were determined in patients with normoglycemic (NG) and Pre-DM. Horizontal lines represent the median values and interquartile ranges. Kruskal–Wallis test. ***, *P* < 0.001. **B** Prevalence of Pre-DM stratified according to TG/HDL-C categories. Linear regression analysis, P for trend  < 0.001

We grouped participants into those with and without Pre-DM. Participants with Pre-DM had an elevated TG/HDL-C ratio level (median 0.8, IQR 0.6–1.3), as compared with NG (median 0.7, IQR 0.5–1.0) (Fig. 4A). In addition, the distribution level of the TG/HDL-C ratio in the Pre-DM group was relatively higher than those in the NG group (Additional file 1: Figure S1).

### The prevalence rate of Pre-DM

Pre-DM occurred in 14970 participants in this study. The prevalence rate of all participants was 9.77% (9.63–9.92%). In addition, the prevalence rates of those five TG/HDL-C ratio groups were 5.79%(5.53–6.05%), 7.51%(7.21–7.80%), 9.04%(8.72–9.36%), 11.24%(10.89–11.60%), and 15.29%(14.89–15.69%), respectively. Participants with higher TG/HDL-C ratios had a higher prevalence of Pre-DM compared to the lowest TG/HDL-C ratio group (*P* < 0.0001 for trend) (Table 2 and Fig. 4B).

**Table 2:** Prevalence rate of Pre-DM

TG/HDL-C ratio	Participants(n)	Pre-DM (n)	Prevalence rate (95%CI) (%)
Total	153163	14970	9.77 (9.63–9.92)
Q1	30631	1773	5.79 (5.53–6.05)
Q2	30631	2300	7.51 (7.21–7.80)
Q3	30626	2768	9.04 (8.72–9.36)
Q4	30631	3444	11.24 (10.89–11.60)
Q5	30644	4685	15.29 (14.89–15.69)
*P* for trend		< 0.001	

### Relationship between TG/HDL-C ratios levels and Pre-DM

The univariate analysis was conducted on the available data, showing that male, age, BMI, ALT, GGT, AST, GLB, TB, Scr, SUA, TC, BUN, TG, LDL-C and TG/HDL-C ratio were positively linked to Pre-DM, while ALB, DBIL and HDL-C were negatively associated with Pre-DM (Additional file 1: Table S1). The multivariate logistic regression analysis showed the association between TG/HDL-C ratios levels and incident Pre-DM. In the crude model, an increase of 1 unit of TG/HDL-C ratio was related to an 79.2% increase in Pre-DM risk (OR = 1.792, 95%CI 1.744–1.842, *P* < 0.001) (Table 3). This relationship of TG/HDL-C ratio and Pre-DM persisted despite adjustment for age, gender, BMI (OR = 1.247, 95%CI 1.208–1.288, *P* < 0.001) (model I) or inclusion of the baseline characteristics and GGT, ALT, ALB, GLB, AST, DBIL, BUN, Scr, UA, TC, LDL-C and TB (model II) (OR = 1.185, 95%CI 1.145–1.226, *P* < 0.001) (Table 3). In addition, when we set the lowest quintile as a reference, the highest quintile of TG/HDL-C ratio was distinctly associated with increased risk for Pre-DM (Crude model, Q5: OR = 2.938, 95%CI 2.744–3.110, *P* < 0.001) (Model I, Q5: OR = 1.497, 95%CI 1.404–1.595, *P* < 0.001) (Model II, Q5: OR = 1.377, 95%CI 1.288–1.473, *P* < 0.001) (Table 3).

**Table 3:** Relationship between TG/HDL-C ratio and Pre-DM in different models

Variable	Crude model (OR,95%CI, P)	Model I (OR,95%CI, P)	Model II (OR,95%CI, P)	Model III (OR,95%CI, P)
TG/HDL-C	1.792 (1.744, 1.842) < 0.001	1.247 (1.208, 1.288) < 0.001	1.185 (1.145, 1.226) < 0.001	1.247 (1.208, 1.288) < 0.001
TG/HDL-C(Quintile)				
Q1	Ref	Ref	Ref	Ref
Q2	1.321 (1.239, 1.409) < 0.001	1.118 (1.045, 1.196) < 0.001	1.101 (1.028, 1.178) < 0.001	1.118 (1.045, 1.196) < 0.001
Q3	1.617 (1.520, 1.720) < 0.001	1.165 (1.091, 1.244) < 0.001	1.129 (1.055, 1.208) < 0.001	1.165 (1.091, 1.244) < 0.001
Q4	2.062 (1.942, 2.189) < 0.001	1.272 (1.193, 1.357) < 0.001	1.205 (1.126, 1.288) < 0.001	1.272 (1.193, 1.357) < 0.001
Q5	2.938 (2.774, 3.110) < 0.001	1.497 (1.404, 1.595) < 0.001	1.377 (1.288, 1.473) < 0.001	1.497 (1.404, 1.595) < 0.001
*P* for trend	< 0.001	< 0.001	< 0.001	< 0.001

Crude model: we did not adjust other covariants

Model I: we adjusted age, sex, BMI

Model II: we adjusted age, sex, BMI, ALT, AST, GGT, ALB, GLB, DBIL, BUN, Scr, UA, TC, LDL-C, TB

Model III: we adjusted age(smooth), sex, BMI(smooth), ALT(smooth), AST(smooth), ALB(smooth), GLB(smooth), DBIL(smooth), BUN(smooth), Scr(smooth), UA(smooth),TC(smooth), LDL-C(smooth), TB(smooth)

*OR* odds ratios, *CI* confidence, *Ref* reference, *TG/HDL-C* triglyceride-to-high-density lipoprotein cholesterol ratio

### Sensitivity analysis

The authors used a GAM to insert the continuity covariate into the equation as a curve with the fully adjusted model (Model III, OR = 1.247, 95%CI 1.208–1.228, *P* < 0.00001) (Table 3). In addition, The authors also excluded participants with LDL-C  < 2.07 mmol/L and TG > 1.7 mmol/L for sensitivity analyses, TG/HDL-C ratio was still positively associated with Pre-DM (Table 4). Furthermore, considering excluding outliers in the TG/HDL-C ratio potentially impacted the association between TG/HDL-C ratio and Pre-DM, the authors also analyzed the association of the TG/HDL-C ratio with Pre-DM in sensitivity analysis without excluding TG/HDL-C ratio outliers (Additional file 1: Table S2). The results obtained from all of the sensitivity analyses indicated the well-robustness of the relationship between TG/HDL-C ratio and Pre-DM.

**Table 4:** Relationship between TG/HDL-C ratio and Pre-DM in different sensitivity analyses

Exposure	Model I (OR,95%CI, P)	Model II (OR,95%CI, P)
TG/HDL-C	1.166 (1.120, 1.214) < 0.001	1.202 (1.119, 1.291) < 0.001
TG/HDL-C (Quintile)		
Q1	Ref	Ref
Q2	1.117 (1.023, 1.219) 0.013	1.089 (1.017, 1.166) 0.015
Q3	1.149 (1.055, 1.251) 0.001	1.110 (1.037, 1.189) 0.003
Q4	1.223 (1.124, 1.330) < 0.001	1.179 (1.100, 1.264) < 0.001
Q5	1.359 (1.250, 1.479) < 0.001	1.246 (1.146, 1.354) < 0.001
*P* for trend	< 0.001	< 0.001

Model I was sensitivity analysis in participants without LDL-C  < 2.07 mmol/L. We adjusted age, sex, BMI, ALT, AST, GGT, ALB, GLB, DBIL, BUN, Scr, UA, TC, LDL-C, TB

Model II was sensitivity analysis in participants without TG  > 1.7 mmol/L. We adjusted age, sex, BMI, ALT, AST, GGT, ALB, GLB, DBIL, BUN, Scr, UA, TC, LDL-C, TB

*OR* odds ratios, *CI* confidence, *Ref* reference, *TG/HDL-C* triglyceride-to-high-density lipoprotein cholesterol ratio

### The nonlinearity addressed by the GAM model

**Fig.5:**
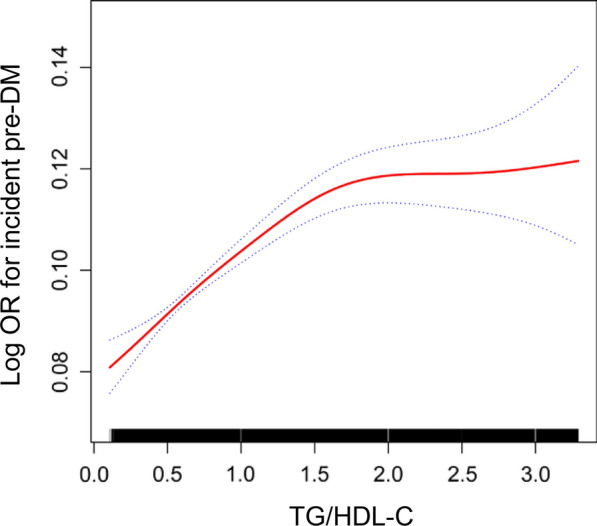
Association of TG/HDL-C with the risk of Pre-DM. The non-linear relationship between TG/HDL-C and incident of Pre-DM. A non-linear relationship between them was detected after adjusting age, sex, BMI, ALT, AST, GGT, ALB, GLB, DBIL, BUN, Scr, UA, TC, LDL-c, TB

GAM and smooth curve fitting were used to study the relationship between TG/HDL-C ratio and Pre-DM. A non-linear relationship between the TG/HDL-C ratio and Pre-DM was detected after adjusting the confounding variables (age, sex, BMI, ALT, AST, GGT, ALB, GLB, DBIL, BUN, Scr, UA, TC, LDL-C, TB) (log likelihood ratio test *P* < 0.001) (Fig. 5). The inflection point of TG/HDL-C ratio were 1.617. On the right of inflection point, the effect size was 1.312 (95%CI 1.242–1.386; *P* < 0.001). However, on the left side of the inflection point, we did not observe a significant association between TG/HDL-C ratio and Pre-DM (OR = 0.980, 95%CI 0.898–1.070; P = 0.658) (Table 5).

**Table 5:** The result of the two-piecewise linear regression model

	Pre-DM (OR,95%CI, *P*)
Fitting model by standard linear regression	1.185 (1.145, 1.226) < 0.001
Fitting model by two-piecewise linear regression	
Inflection point of TG/HDL-C	1.617
≤ 1.617	1.312 (1.242, 1.386) < 0.001
> 1.617	0.980 (0.898, 1.070) 0.658
*P* for the log-likelihood ratio test	< 0.001

We adjusted age, sex, BMI, ALT, AST, GGT, ALB, GLB, DBIL, BUN, Scr, UA, TC, LDL-C, TB

*OR* odds ratios, *CI* confidence, *Ref* reference, *TG/HDL-C* triglyceride-to-high-density lipoprotein cholesterol ratio

### The results of subgroup analyses

In Table 6 showed that gender, age, gender, BMI, and ALT could modify the relationship between TG/HDL-C ratio and Pre-DM (All P for interaction < 0.05). And a stronger association was observed in females (OR = 1.366, 95%CI 1.287–1.450) and the population with ALT ≤ 40 U/L (OR = 1.190, 95%CI 1.149–1.232), and BMI between 18.5 and 24 kg/m^2^ (OR = 1.253, 95%CI 1.206–1.301). A stronger relationship could also be observed among those participants between the ages of 30 and 40 (OR = 1.265, 95%CI 1.168–1.369) and those between the ages of 40 and 50 (OR = 1.238, 95%CI 1.158–1.323). In contrast, the weaker association was probed in males(OR = 1.109, 95%CI 1.065–1.155), and the population with ALT ≥ 40U/L (OR = 1.190, 95%CI 1.149–1.232), BMI ≥ 24 kg/m^2^(OR = 1.153, 95%CI 1.066–1.247), and those younger 30 years (OR = 1.169, 95%CI 1.011–1.353). There was no relationship between TG/HDL-C ratio and Pre-DM in those over 60 years old or with the BMI < 24 kg/m^2^.

**Table 6:** Effect size of TG/HDL-C on Pre-DM in prespecified and exploratory subgroups

Characteristic	No of participants	OR (95%CI)	*P* value	*P* for interacion
Age, years				< 0.001
< 30	40094	1.169 (1.011, 1.353)	0.036	
30–40	50062	1.265 (1.168, 1.369)	< 0.001	
40–50	32980	1.238 (1.158, 1.323)	< 0.001	
50–60	15748	1.145 (1.066, 1.230)	< 0.001	
60–70	6767	1.000 (0.907, 1.102)	0.999	
≥ 70	7512	1.057 (0.963, 1.161)	0.240	
Gender				< 0.001
Female	85861	1.366 (1.287, 1.450)	< 0.001	
Male	67302	1.109 (1.065, 1.155)	< 0.001	
BMI (kg/m^2^)				0.012
< 18.5	18005	0.928 (0.726, 1.187)	0.552	
≥ 18.5, < 24	121856	1.253 (1.206, 1.301)	< 0.001	
≥ 24	13302	1.153 (1.066, 1.247)	0.004	
ALT (U/L)				0.005
≤ 40	144562	1.296 (1.138, 1.475)	< 0.001	
> 40	8601	1.190 (1.149, 1.232)	< 0.001	
GLB (g/L)				0.348
< 30	89724	1.202 (1.148, 1.258)	< 0.0001	
≥ 30	63439	1.163 (1.105, 1.224)	< 0.0001	

Note 1: Above model adjusted for age, sex, BMI, ALT, AST, GGT, ALB, GLB, DBIL, BUN, Scr, UA, TC, LDL-C, TB

Note 2: In each case, the model is not adjusted for the stratification variable

*OR* odds ratios, *CI* confidence, *Ref* reference, *TG/HDL-C* triglyceride-to-high-density lipoprotein cholesterol ratio

## Discussion

This cross-sectional study described an elevated level of TG/HDL-C ratio was related to the increased risk of Pre-DM in a Chinese non-obese population with a normal range of LDL-C. There was a non-linear relationship between TG/HDL-C ratio and Pre-DM, which the inflection point was 1.617. When the TG/HDL-C ratio level  < 1.617, we found a strong positive correlation between TG/HDL-C ratio level and Pre-DM (OR = 1.312, 95% CI 1.242–1.386). In addition, age, sex, BMI, ALT were found as the potential effect modifiers to modify the relationship between TG/HDL-C ratio and Pre-DM, as significantly stronger associations were observed in females and the population with 30 years  < age  < 40 years, 18.5 kg/m^2^ < BMI < 24 kg/m^2^, and ALT < 40U/L. The above findings indicated that TG/HDL-C ratio may provide a valuable reference for the primary prevention of Pre-DM in a Chinese non-obese population with a normal range of LDL-C.

Pre-DM affects about 35.7% of adults in China [2]. However, the prevalence of Pre-DM in the present study was only 9.77%, lower than the prevalence of general population. The acceptable reason was that the participants in this study excluded the obese participants and those with LDL-C  > 3.12 mmol/L, both of which are risk factors for Pre-DM. Notably, even in non-obese participants with normal LDL-C range, the prevalence of Pre-DM was still 9.77%, especially in men. Therefore, it was still imperative to look for the risk factors leading to Pre-DM. The goal of screening for the risk factors leading to Pre-DM and treat these conditions earlier rather than later to prevent progression of disease and adverse outcomes.

Compared the traditional lipid parameters including TG, TC, HDL-C, LDL-C, non-traditional lipid parameters like TG/HDL-C, LDL-C/HDL-C, non-HDL-C, TC/HDL-C and non-HDL-C/HDL-C are closely related to the occurrence of pre-DM. Especially TG/HDL-C, which has been recognized as a biomarker of insulin resistance that may accelerate the development of prediabetes, is more effective than single lipids measures [11]. Similar results were observed in individuals with normal BMI and LDL-c in this present study. Fahim et al. and Leay-Kiaw et al. compared different surrogate estimates of insulin action with insulin concentration in nondiabetic individuals and revealed that calculation of TG/HDL-C ratio and triglyceride-glucose index were correlated with insulin resistance to a similar degree [17, 18]. However, in non-obese individuals with normal LDL-C levels in this study, the receiver operator characteristic analysis showed that the TG/HDL-C had the highest predictive value compared with TyG and traditional lipid parameters (data not show).

The following reasons may explain the relationship of TG/HDL-C ratio with pre-DM. A higher concentration of TGs leads to the development of pre-DM mainly via FFAs [11]. Elevated TG results in increased free fatty acids (FFAs), leading to the production of toxic lipids, which lead to change in pancreatic α-cell insulin signaling and glucagon hypersecretion [19, 20]. High glucagon levels are thought to be a major contributor to hyperglycemia in diabetics. Plasma glucagon stimulates hepatic glucose output by promoting glycogenolysis and gluconeogenesis [21]. At the same time, HDL plays a role in the regulation of pancreatic islet cell secretory function and apoptosis. Previous study identified two components of HDL, ApoA1 and S1P, by which HDL protect β-cells from cytokine- or glucose-induced apoptosis [22].Decreased HDL-C levels affect the β-cell, which has an important role in the pathogenesis of Pre-DM [22, 23]. Therefore, dyslipidemia predicted the onset of Pre-DM. Furthermore, insulin regulates the rate-limiting enzymes involved in lipolysis, as well as in lipid synthesis, by decreasing their serine/threonine phosphorylation state via a combination of protein kinase inhibition and phosphatase activation [24, 25]. The insulin-mediated control of sterol-regulatory element binding protein expression in the liver and adipose tissue is central to alterations in lipid metabolism associated with hyperinsulinemia, over-activity of sterol-regulatory element binding protein signaling may lead to dyslipidemia [26]. Therefore, insulin plays a key role in the control of hepatic lipid metabolism and the development of hepatic steatosis during insulin resistance [27].

In subgroup analysis, TG/HDL-C ratio was associated with pre-DM at different ages, sexes, BMI and ALT, but stronger in female and the population with 30 years  < age  < 40 years, 18.5 kg/m^2^ ≤ BMI < 24 kg/m^2^, and ALT ≤ 40U/L. Since these factors could modify the relationship between TG/HDL-C ratio and Pre-DM, it is clinically possible to reduce the risk of Pre-DM by altering the strength of the association between the TG/HDL-C ratio and Pre-DM through interfering with BMI and ALT levels.

Furthermore, the present study observed a non-linear relationship between TG/HDL-C ratio and Pre-DM for the first time, which the inflection point was 1.617 after adjusting for confounders. When the TG/HDL-C ratio was  ≤ 1.617, 1 unit increase in TG/HDL-C ratio is accompanied by a 31.2% increase for the incidence Pre-DM (OR = 1.312, 95% CI 1.242–1.386). However, when the TG/HDL-C ratio was  > 1.617, the TG/HDL-C ratio was no correlation with incident Pre-DM (OR = 0.980, 95% CI 0.898–1.070). The reason may be that other variables other than the TG/HDL-C ratio also affected Pre-DM. It could be seen from Table S4 that compared with the TG/HDL-C ratio  ≤ 1.617, participants with TG/HDL-C ratio  > 1.617 have generally higher levels of BMI, TC, LDL-C, and a higher proportion of males. However, the above indicators were closely related to Pre-DM [8]. When TG/HDL-C ratio greater than 1.617, due to the presence of these Pre-DM risk factors, TG/HDL-C ratio had a relatively weak effect on Pre-DM risk. On the contrary, when TG/HDL-C ratio was less than 1.617, the level of the risk factors for Pre-DM, such as BMI, FPG, TC, LDL-C was lower, and the impact on Pre-DM was weakened, at this time the effect of TG/HDL-C ratio was relatively enhanced (Additional file 1: Table S4). The inflection point is expected to provide evidence for the clinicians to manage TG/HDL-C ratio. From a treatment perspective, it makes sense to reduce the TG/HDL-C ratio level below the inflection point, which can significantly reduce the risk of progression to Pre-DM.

Compared to other studies, this study has the following three works add to existing knowledge. Firstly, previous study showed that in the 116,855 Chinese general participants from 32 locations, the higher incidence of prediabetes was significantly associated with the higher TG/HDL (OR = 1.04, 95% CI 1.02–1.06) [28]. In addition, Guo et al. demonstrated that a positive relationship between the TG/HDL-c and the risk of prediabetes in Chinese patients with a mean TG/HDL ratio of 1.74 ± 0.90, a mean BMI level of 25.82 ± 2.98 kg/m [2] and a mean LDL-c level of 3.12 ± 0.81 mmol/L [9]. In the present cross-sectional study, having a larger sample size including 153,163 participants, the logistic regression model showed a positive association between the TG/HDL-C ratio and the risk of Pre-DM, consistent with those two studies. However, compared with the other two studies, BMI, LDL-C levels and TG/HDL-C ratio levels were lower in the present cross-sectional study (Table 2, Additional file 1: Table S3). The reason is that the present study was investigated in different populations, which limit the population to non-obese participants with normal LDL-c range. Abnormal BMI and LDL-c are risk factors for pre-DM. However, previous study showed that a significant proportion (13%) in the study of 254 non-obese young adults had prediabetes [10]. Participants with normal LDL-c range could also lead to pre-DM [9]. Early attention and intervention are taken in obese and dyslipidemia participants in order to reduce the incidence of prediabetes. However, non-obese participants with normal LDL-c ranges are often overlooked. Since prediabetes appears to be prevalent in non-obese adults and participants with normal LDL-c range, it is critical to screen risks factors for this population. To our knowledge, the association between TG/HDL-c and pre-DM in the non-obese population with normal blood lipid levels has not been reported. Thus, the aim of this study was to develop a deeper understanding of the relationship of TG/HDL-c and pre-DM in non-obese individuals with normal LDL-c. Secondly, compared to the previous research, addressing non-linearity is a significant improvement, which provided a reference for the management of TG/HDL-C ratio in the non-obese Chinese population with normal LDL-C range. Moreover, the authors adjusted more biochemical parameters in the present study, including ALB, GLB, DBIL, and BUN. Evidence showed that those parameters were associated with Pre-DM [29–32]. In testing the robustness of the results by a set of sensitivity analyses (target independent variable transformation, subgroup analysis and using a GAM to insert the continuity covariate into the equation as a curve), the stronger positive association was found in female and the population with 30 years  < age  < 40 years, 18.5 kg/m^2^ ≤ BMI < 24 kg/m^2^, and ALT ≤ 40U/L, which would be the spotlight of attention in clinic. The efforts as mentioned above have confirmed the relationship's stability between TG/HDL-C ratio and Pre-DM risk in non-obese population with normal LDL-c range. Thirdly, the results provided a reference for clinical intervention in TG/HDL-C ratio levels to reduce the risk of Pre-DM risk in non-obese population with normal LDL-c range. The findings suggested the potential for a substantial advantage for pre-DM of improving lipoprotein metabolism in the non-obese population with normal LDL-c. Early intervention may improve the outcome if more lifestyle or therapeutic efforts to decrease the TG/HDL ratio are focused on the early stages.

### Study strength and limitations

The present study has some advantages, and we listed them as follows. Firstly, this is the first time to observe the association between TG/HDL-C ratio and Pre-DM in Chinese non-obese people with a normal range of low-density lipoprotein cholesterol with a large sample size. It provides a rationale for screening and taking more attention of the prevalence of prediabetes on healthy persons. Secondly, the non-linear relationship between TG/HDL-C ratio and Pre-DM was firstly discovered and the inflection point was calculated. Thirdly, the robustness of this study was tested with a set of sensitivity analyses (target independent variable transformation, subgroup analysis and using a GAM to insert the continuity covariate into the equation as a curve). However, there are some limitations of this study. Firstly, the findings of this study can be generalized to Chinese non-obese people with a normal range of LDL-C only, which might be different in participants with participants over 60 years old or BMI < 24 kg/m^2^. Secondly, as with all observational studies, although known potential confounders such as blood pressure, ALT were controlled, there may still be uncontrolled or unmeasured confounders.

## Conclusions

This study demonstrates a positive and non-linear relationship between TG/HDL-C ratio and incident Pre-DM in the Chinese non-obese people with a normal range of LDL-C. There was a threshold effect between the TG/HDL-C ratio level and Pre-DM. When TG/HDL-C ratio is less than 1.617, it is positive correlated with the incident Pre-DM. This result is expected to provide reference for the clinicians to control TG/HDL-C ratio. Reducing the TG/HDL-C ratio level below 1.617 can significantly reduce the risk of progression to Pre-DM. Thus, abnormal TG/HDL-C ratio supports identifying the high risk of Pre-DM in Chinese non-obese population with a normal range of LDL-C, which would help clinicians plan and initiate the effective way to prevent Pre-DM in advance.

## Supplementary Information

**Additional file 1: Table S1. **The results of univariate analysis. **Table S2.** Relationship between TG/HDL-C ratio and Pre-DM in participants without excluding outliers of TG/HDL-C ratio. **Table S3.** The characteristics of participants between NG and Pre-DM groups. **Table S4. **The characteristics of participants on both sides of the inflection point. **Figure S1.** The TG/HDL-C ratio levels of all participants from the Pre-DM and NG groups. The distribution level of the TG/HDL-C ratio in the Pre-DM group was higher than the NG groups.

## Data Availability

Data can be downloaded from ‘DATADRYAD’ database (www.Datadryad.org).

## Abbreviations

ALT: Alanine aminotransferase
ALB: Albumin
AST: Aspartate aminotransferase
BMI: Body mass index
BUN: Serum urea nitrogen
BP: Blood pressure
CI: Confidence intervals
DBIL: Direct bilirubin
DM: Diabetes
FFAs: Free fatty acids
FPG: Fasting plasma glucose
GAM: Generalized additive model
GLB: Globulin
GGT: γ-Glutamyl transpeptidase
HDL-c: High-density lipoprotein cholesterol
IQR: Interquartile ranges
LDL-c: Low-density lipoprotein cholesterol
NAFLD: Non-alcoholic fatty liver disease
OR: Odds ratio
pre-DM: Prediabetes
SD: Standard deviation
Scr: Serum creatinine
TC: Total cholesterol
TG: Triglyceride
TB: Total bilirubin
T2DM: Type 2 diabetes mellitus
TG/HDL-C: Triglyceride-to-high-density lipoprotein cholesterol
UA: Uric acid
