# Supplementary material for: Association between triglyceride-to-high-density lipoprotein cholesterol ratio and prediabetes: a cross-sectional study in Chinese non-obese people with a normal range of low-density lipoprotein cholesterol
Source: J Transl Med. 2022 Oct 22;20:484. doi: 10.1186/s12967-022-03684-1 (PMC9588227; doi:10.1186/s12967-022-03684-1)
Supplement: Supplementary file 1 — Additional file 1: Table S1. The results of univariate analysis. Table S2. Relationship between TG/HDL-C ratio and Pre-DM in participants without excluding outliers of TG/HDL-C ratio. Table S3. The characteristics of participants between NG and Pre-DM groups. Table S4. The characteristics of participants on both sides of the inflection point. Figure S1. The TG/HDL-C ratio levels of all participants from the Pre-DM and NG groups. The distribution level of the TG/HDL-C ratio in the Pre-DM group was higher than the NG groups. [file 12967_2022_3684_MOESM1_ESM.pdf]

Supplementary data

Association between triglyceride-to-high-density lipoprotein cholesterol ratio and prediabetes: a cross-sectional study in Chinese non-obese people with a normal range of low-density lipoprotein cholesterol

Running title: The relationship between TG/HDL-C ratio and Pre-DM

Liling Wu[1,2†],Xiaodan Wu[3†] , Haofei Hu[1,2*], Qijun Wan[1,2*]

[1]Department of Nephrology, The First Affiliated Hospital of Shenzhen University, Shenzhen 518000, Guangdong Province, China.

[2]Department of Nephrology, Shenzhen Second People's Hospital, Shenzhen 518000, Guangdong Province, China.

[3]Department of Anesthesiology, Nanfang Hospital, Southern Medical University, Guangzhou, Guangdong 510515, P.R.China

Full list of author information is available at the end of the article.

[*]Correspondence: yiyuan2224@sina.com and huhaofei0319@126.com.

[†]Liling Wu and Xiao-dan Wu contributed equally to this work.

Total number of tables: 4

Total number of figure: 1

Table S1. The results of univariate analysis

Variable	Statistics	OR (95%CI)	*P* value
**Age, years**	39.591 ± 13.689	1.062 (1.061, 1.064)	<0.001
**Gender**			
**Female**	85861 (56.059%)	Ref.	
**Male**	67302 (43.941%)	2.254 (2.177, 2.334)	<0.001
**BMI, kg/m^2**	21.074 ± 2.069	1.267 (1.256, 1.279)	<0.001
**GGT,U/L**	25.317 ± 23.627	1.009 (1.009, 1.010)	<0.001
**ALT,U/L**	18.804 ± 17.308	1.003 (1.003, 1.004)	<0.001
**AST,U/L**	22.241 ± 11.084	1.008 (1.007, 1.010)	<0.001
**ALB,g/L**	44.522 ± 3.019	0.988 (0.983, 0.994)	<0.001
**GLB,g/L**	29.297 ± 3.785	1.032 (1.027, 1.036)	<0.001
**TB, umol/L**	12.086 ± 5.061	1.008 (1.005, 1.011)	<0.001
**DBIL, umol/L**	2.008 ± 1.041	0.968 (0.952, 0.984)	<0.001
**BUN,mmol/L**	4.344 ± 1.269	1.317 (1.301, 1.333)	<0.001
**Scr, umol/L**	77.374 ± 21.885	1.016 (1.015, 1.017)	<0.001
**UA, umol/L**	271.415 ± 83.919	1.003 (1.003, 1.003)	<0.001
**TC, mmol/L**	4.484 ± 0.719	1.428 (1.395, 1.462)	<0.001
**TG, mmol/L**	1.121 ± 0.538	1.873 (1.825, 1.923)	<0.001
**HDL-C, mmol/L**	1.490 ± 0.348	0.708 (0.673, 0.745)	<0.001
**LDL-C, mmol/L**	2.222 ± 0.470	1.781 (1.715, 1.848)	<0.001
**TG/HDL-C**	0.824 ± 0.518	1.792 (1.744, 1.842)	<0.001

Values are n(%) or mean±SD

ALB, albumin; ALT, alanine aminotransferase; AST, aspartate aminotransferase; BMI, body mass index; BUN, blood urea nitrogen; Scr, serum creatinine; DBIL, direct bilirubin; TB, total bilirubin; GGT, γ-glutamyl transpeptidase; GLB, globulin; HDL-C, high-density lipoprotein cholesterol; LDL-C, low-density lipoprotein cholesterol; TC, total cholesterol; TG, triglyceride; UA, uric acid; TG/HDL-C: triglyceride-to-high-density lipoprotein cholesterol ratio

Table S2. Relationship between TG/HDL-C ratio and Pre-DM in participants without excluding outliers of TG/HDL-C ratio

Variable	Crude model (OR,95%CI, P)	Model I(OR,95%CI, P)	Model II (OR,95%CI, P)
**TG/HDL-C**	1.710 (1.688, 1.732) <0.001	1.569 (1.546, 1.592) <0.001	1.334 (1.312, 1.356) <0.001
**TG/HDL-C (Quintile)**			
**Q1**	1.0	1.0	1.0
**Q2**	1.616 (1.526, 1.710) <0.001	1.384 (1.302, 1.471) <0.001	1.275 (1.196, 1.359) <0.001
**Q3**	2.381 (2.256, 2.512) <0.001	1.849 (1.745, 1.960) <0.001	1.592 (1.498, 1.693) <0.001
**Q4**	3.387 (3.216, 3.568) <0.001	2.492 (2.356, 2.637) <0.001	1.933 (1.821, 2.052) <0.001
**Q5**	5.615 (5.341, 5.903) <0.001	3.947 (3.737, 4.169) <0.001	2.548 (2.402, 2.702) <0.001
***P* for trend**	<0.001	<0.001	<0.001

Crude model: we did not adjust other covariants

Model I: we adjusted age, sex, BMI

Model II: we adjusted age, sex, BMI, ALT, AST, GGT, ALB, GLB, DBIL, BUN, Scr, UA, TC, LDL-C, TB

OR, odds ratios; CI: confidence, Ref: reference; TG/HDL-C: triglyceride-to-high-density lipoprotein cholesterol ratio

Table S3. The characteristics of participants between NG and Pre-DM groups.

	NG	Pre-DM	*P*-value
**N**	138193	14970	
**Age(years)**	38.2 ± 12.7	52.3 ± 15.9	<0.001
**Gender**			<0.001
**Female**	80172 (58.0%)	5689 (38.0%)	
**Male**	58021 (42.0%)	9281 (62.0%)	
**BMI(kg/m^2)**	21.0 ± 2.1	21.9 ± 1.9	<0.001
**GGT(U/L)**	19.0 (14.0-28.4)	23.0 (17.0-35.0)	<0.001
**ALT(U/L)**	15.0 (11.0-22.5)	17.0 (13.0-23.0)	<0.001
**AST(U/L)**	20.0 (17.0-25.0)	22.0 (19.0-26.0)	<0.001
**ALB(g/L)**	44.5 ± 3.0	44.4 ± 3.0	<0.001
**GLB(g/L)**	29.3 ± 3.8	29.7 ± 4.0	<0.001
**TB(umol/L)**	12.1 ± 5.1	12.3 ± 5.0	<0.001
**DBIL(umol/L)**	1.9 (1.3-2.6)	1.8 (1.3-2.5)	0.0004
**BUN(mmol/L)**	4.3 ± 1.2	4.8 ± 1.5	<0.001
**Scr(umol/L)**	76.6 ± 20.2	84.8 ± 33.0	<0.001
**SUA(umol/L)**	269.3 ± 83.4	291.4 ± 86.5	<0.001
**FPG(mmol/L)**	4.9 ± 0.3	6.0 ± 0.3	<0.001
**TC(mmol/L)**	4.5 ± 0.7	4.7 ± 0.7	<0.001
**TG(mmol/L)**	1.1 ± 0.5	1.3 ± 0.6	<0.001
**HDL-C(mmol/L)**	1.5 ± 0.3	1.5 ± 0.4	<0.001
**LDL-C(mmol/L)**	2.2 ± 0.5	2.3 ± 0.5	<0.001
**TG/HDL-C**	0.7 (0.5-1.0)	0.8 (0.6-1.3)	<0.001

Values are n (%) or mean ± SD or median (quartile)

ALB, albumin; ALT, alanine aminotransferase; AST, aspartate aminotransferase; BMI, body mass index; BUN, blood urea nitrogen; Scr, serum creatinine; DBIL, direct bilirubin; TB, total bilirubin; FPG, fasting plasma glucose; GGT, γ-glutamyl transpeptidase; GLB, globulin; HDL-C, high-density lipoprotein cholesterol; LDL-C, low-density lipoprotein cholesterol; TC, total cholesterol; TG, triglyceride; UA, uric acid; TG/HDL-C: triglyceride-to-high-density lipoprotein cholesterol ratio

Table S4. The characteristics of participants on both sides of the inflection point.

TG/HDL-C ratio	<1.617	>=1.617	*P*-value
**N**	140439	12724	
**Age(years)**	39.2 ± 13.6	44.0 ± 14.0	<0.001
**Gender**			<0.001
**Female**	82902 (59.0%)	2959 (23.3%)	
**Male**	57537 (41.0%)	9765 (76.7%)	
**BMI(kg/m^2)**	21.0 ± 2.1	22.4 ± 1.7	<0.001
**GGT(U/L)**	19.0 (14.0-28.0)	28.0 (20.0-42.0)	<0.001
**ALT(U/L)**	15.0 (11.0-22.0)	20.0 (14.0-28.0)	<0.001
**AST(U/L)**	20.0 (17.0-25.0)	22.0 (19.0-26.0)	<0.001
**ALB(g/L)**	44.5 ± 3.0	44.9 ± 2.9	<0.001
**GLB(g/L)**	29.3 ± 3.8	29.4 ± 3.8	0.017
**TB(umol/L)**	12.1 ± 5.1	12.1 ± 5.1	<0.001
**DBIL(umol/L)**	1.9 (1.3-2.6)	1.7 (1.2-2.4)	<0.001
**BUN(mmol/L)**	4.3 ± 1.3	4.5 ± 1.4	<0.001
**Scr(umol/L)**	76.6 ± 20.7	86.4 ± 30.5	<0.001
**UA(umol/L)**	265.6 ± 81.3	335.5 ± 86.1	<0.001
**FPG(mmol/L)**	5.0 ± 0.5	5.2 ± 0.5	0.190
**TC(mmol/L)**	4.5 ± 0.7	4.7 ± 0.7	<0.001
**TG(mmol/L)**	0.9 (0.7-1.2)	2.2 (1.9-2.6)	<0.001
**HDL-C(mmol/L)**	1.5 ± 0.3	1.1 ± 0.2	<0.001
**LDL-C(mmol/L)**	2.2 ± 0.5	2.4 ± 0.4	<0.001
**TG/HDL-C**	0.6 (0.5-0.9)	2.0 (1.8-2.4)	<0.001

Values are n (%) or mean ± SD or median (quartile)

ALB, albumin; ALT, alanine aminotransferase; AST, aspartate aminotransferase; BMI, body mass index; BUN, blood urea nitrogen; Scr, serum creatinine; DBIL, direct bilirubin; TB, total bilirubin; FPG, fasting plasma glucose; GGT, γ-glutamyl transpeptidase; GLB, globulin; HDL-C, high-density lipoprotein cholesterol; LDL-C, low-density lipoprotein cholesterol; TC, total cholesterol; TG, triglyceride; UA, uric acid; TG/HDL-C: triglyceride-to-high-density lipoprotein cholesterol ratio

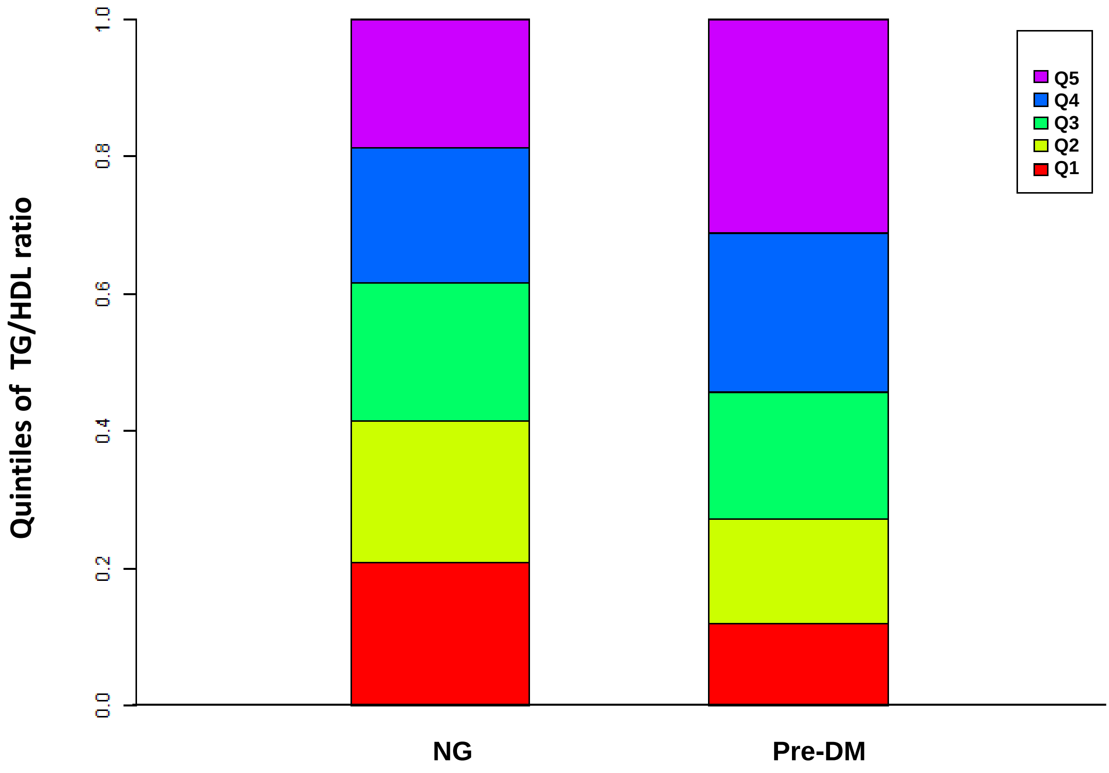

Figure S1. The TG/HDL-C ratio levels of all participants from the Pre-DM and NG groups

The distribution level of the TG/HDL-C ratio in the Pre-DM group was higher than the NG groups
